# The benefits of fully electronic consent management and consent collection via a tablet PC in supporting time-critical pandemic research—an example from a NAPKON COVID-19 project

**DOI:** 10.3389/fdgth.2025.1489176

**Published:** 2025-09-29

**Authors:** Dana Stahl, Henriette Rau, Arne Blumentritt, Lizon Fiedler-Lacombe, Ekaterina Heim, Heike Valentin, Martin Bialke, Monika Kraus, Wolfgang Hoffmann

**Affiliations:** ^1^ Trusted Third Party of the University Medicine Greifswald, Greifswald, Germany; ^2^ Institute for Community Medicine, Section Epidemiology of Health Care and Community Health, University Medicine Greifswald, Greifswald, Germany; ^3^ Institute of Epidemiology, Helmholtz Munich, Munich, Germany

**Keywords:** COVID-19, recruitment, informed consent, electronic consent, study inclusion

## Abstract

**Objective:**

A study participant's informed consent, based on study information and expressed using a consent form (CF), is the ethical and legal basis for research with humans. Timely automatic access to a participant’s consent status in different systems is crucial for knowing which medical data, images, and biological samples can be collected for research. To support time-critical (pandemic) research, this article evaluates a fully electronic consent management system and a consent collection process using a tablet PC in comparison to traditional paper-and-pencil-based approaches and assesses their impact on patient recruitment.

**Materials and methods:**

The evaluation is based on a COVID-19 study [the *Sektorenübergreifende Plattform* (SÜP) study; 2,753 study participants] that offered both paper-and-pencil- and tablet-based consent collection approaches and focused on the following: (a) initial CF validity and its impact on patient recruitment, (b) time-to-initial availability of structured consent information for other systems, (c) time-to-research based on completed quality assurance of CFs, and (d) feedback on both approaches from study staff and participants.

**Results:**

The initial CF validity increased significantly from 67.38% for paper-and-pencil-based CFs to 99.46% for tablet-based CFs. This quality increase also reduced the number of invalid CFs or CFs requiring corrections, which can lead to study exclusion and, consequently, lower recruitment rates and lost research data. The time lag between recruitment and the availability of data decreased significantly when using tablet-based CFs, supporting time-critical research while protecting participants’ privacy. Overall, the participants’ and study staff's feedback on tablet-based CF collection was positive and highlighted the benefits of tablet-based CF collection in reducing the documentational burden on study staff and enabling participants to adjust the CF’s appearance, for example, by choosing a bigger font size.

**Discussion:**

Although tablet-based CF collection has measurable positive effects, especially on patient recruitment rates due to an increase in initially valid CFs, the majority of the National Pandemic Cohort Network (German: *Nationales Pandemie Kohorten Netz*, NAPKON) study sites still solely use paper-and-pencil-based processes. Since the feedback from study staff and participants was mainly positive, other barriers beyond technical availability and workflows likely exist and need to be evaluated in further settings.

**Conclusion:**

Fully electronic informed consent collection is the “best practice” approach to ensure valid CFs and increase initial patient inclusion rates in studies. Due to the additional benefits, including shorter time-to-research, electronic consent form collection should be integrated into pandemic response schemes and other time-critical research.

## 1 Introduction

The spread of new (infectious) diseases worldwide, such as COVID-19, requires studies to be rapidly initiated and conducted to address urgent medical questions and provide evidence for healthcare management. In pandemic situations, especially, research involves a variety of (inter-)national partners, such as healthcare providers, pharmaceutical companies, and biomedical institutes that collect, transfer, process, and store data originating from several different sources, e.g., medical procedures, imaging, or biological samples. In such a complex research situation, compliance with relevant legal and ethical regulations can be challenging. One major prerequisite for research involving human subjects is informed consent (IC). Informed consent means that human subjects have been given information about the details of a study and the possible consequences of participating, and that they have had the opportunity to ask questions and discuss this information with qualified study staff. The aim is to ensure that the information given is understood before the eligible study participant agrees to participate in a study. Most research situations require that the participant fills in and signs a consent form (CF) before data can be collected (1–4). Since research participants have the right to privacy and informational self-determination, and personal medical data are seen as sensitive data that must be especially protected, legally valid IC provided by a human subject is currently emphasized as the basis for medical research by numerous declarations and legal frameworks (2, 5–8).

In this article, the term “informed consent” is defined as a study participant's written decision regarding the use of their personal health information, imaging, or biological samples for research purposes (as opposed to use for treatment, reimbursement, or other purposes). The participant's decision on what healthcare providers, researchers, and associated partners are allowed to do with their personal health data and, if applicable, biological samples or imaging, must be unambiguously expressed and clearly stated in the CF (3, 4). For the context of this article, a written form is defined as a participant filling in either a paper or digital form and signing it manually. The validity of CFs should be carefully evaluated (for the definitions of the validity criteria, see Section 2.5). An invalid CF requires re-contacting the potential study participant and re-consenting, or study exclusion and/or deletion of the participant's datasets. Thus, this directly influences work volume and study flow and, consequently, recruitment rates.

Usually, IC from an eligible study participant is collected using a paper form, which is signed in writing by the participant. However, paper-and-pencil-based processes do not readily provide structured and machine-readable information that can be exchanged instantly between all systems involved in research data solicitation and management, e.g., hospital information systems (HISs), picture archiving and communication systems (PACSs), or laboratory information systems (LIMSs). Timely automatic access to the current IC status of each participant in these systems is crucial to know which medical data, images, and biological samples can be collected for research. Therefore, paper-based CFs need to be digitized to obtain structured data. Poor-quality paper-based forms (9), which must be manually corrected prior to digitization, lead to an increased burden on study staff during patient recruitment and delays in the research process. This is a major disadvantage, especially in pandemic-related and time-critical research, such as during the COVID-19 pandemic, and particularly when various service providers and data sources are involved (e.g., clinicians, medical care centers, and laboratories).

Consequently, computer-assisted recruitment and IC assessment are needed. According to MITRE (10), electronic informed consent management was uncommon in 2014, and even today it is still an exception in (inter-)national medical research projects. However, fully digitalized IC management is a key success factor for securing privacy and supporting clinical data sharing on national and international levels in pandemic research and responses. Hence, a portable, adaptable, and fully electronic IC management service is needed that can be readily implemented in recruitment processes across healthcare providers and in different sectors of the healthcare system.

This article evaluated the application of a novel fully electronic consent collection approach for clinical research using an electronic CF that is filled in by the participant via a tablet computer (tablet-based CF) in comparison to the traditional paper-and-pencil method. For this evaluation, a COVID-19 study that used both paper-and-pencil-based and tablet-based consent collection approaches is used as an example.

This article aims to answer the following research questions:

(1) Can fully electronic consent collection and management support pandemic and other time-critical clinical research by
  a) improving CF quality and mitigating its impact on patient inclusion in a clinical study,
  b) reducing time-to-initial availability of structured consent information in different systems (e.g., HISs, PACSs, and LIMSs), and
  c) reducing the time-to-possible-research by reducing the time needed to complete the quality assurance (QA) process for the CFs?
(2) What are the strengths and limitations of the concept according to feedback from study staff and participants on both approaches?

## 2 Materials and methods

### 2.1 Study design, setting, recruitment, and inclusion criteria

The evaluation is based on the COVID-19 cohort study named Sektorenübergreifende Plattform (SÜP) (English: “cross-sector platform”), which was part of the National Pandemic Cohort Network (German: *Nationales Pandemie Kohorten Netz*, NAPKON) (11) for COVID-19 research. The SÜP participants were recruited in university hospitals, non-university hospitals, medical practices, and medical care centers, in a joint effort for COVID-19 research.

SÜP recruited SARS-CoV-2-positive study participants and persons who were SARS-CoV-2-negative as a control cohort. The inclusion criteria for the study participants were a positive polymerase chain reaction (PCR) test that detected SARS-CoV-2 and enrollment within 1 week after a positive PCR test. Medical staff in university and non-university clinics, medical practices (including GPs), and care centers actively invited eligible persons to participate in the SÜP cohort. The study participants received written information that described in detail how and which data would be collected, processed, and used scientifically (2). To provide informed consent, eligible persons filled out and signed either a paper-based CF or an electronic CF using a tablet PC. Both CF approaches had identical content.

Study participants were excluded if their CF was not signed, if their choice was not unambiguously stated, or if the paper form was not filled in correctly and the CF was not corrected within a certain amount of time.

### 2.2 CF structure and management

All the SÜP-CFs were designed as modular CFs (12). This means that following introductory text that informs the participant about SÜP and the privacy policy in general, multiple modules (obligatory and optional) are offered that specifically address dimensions of the study, e.g., medical data, imaging data, and biological samples. At the end of the form, the signatures of the study participant and the medical professional are requested. Optional modules mean that a study participant can refuse or consent to their data being used for several specific purposes, e.g., whether health insurance data can be requested or whether the participant agrees to being re-contacted for defined purposes (for more information, refer to Appendix A). Study participants can indicate their choice for each module individually by ticking either “yes” (consent) or “no” (refusal). Two versions of the consent form were available to the study sites, one with the biomaterial collection option and one without, depending on the capabilities and requirements of the respective study site. Modified CF versions were available to meet the specific requirements of individual federal states or universities.

All the SÜP-CFs were managed by the Trusted Third Party of the University Medicine Greifswald (TTP) using the generic Informed Consent Service (gICS®) (12–15), a free-of-charge and open-source (AGPLv3) software solution (15). The term “management” refers to the generation of both the CF templates that are presented on a tablet or printed and the specific dataset items from the filled-in and signed CFs. The gICS® was integrated into both consent processes and supports paper-based and tablet-based (fully electronic) CF collection. For this, the gICS® provides interfaces for software that manage (study-) pseudonyms [e.g., gPAS (16)] and person-identifying data, including record linkage [e.g., E-PIX (17)]. In addition, interfaces for the SÜP’s infrastructural partners, such as Clinical Data Management (medical data), the imaging data management system, and the LIMS, have been implemented and used since November 2020.

### 2.3 Classification of recruitment workflows and the steps necessary to provide structured and machine-readable consent information

The goal of IC management in complex research projects that integrate various data sources and systems is to provide readily available, structured, and machine-readable information on the consent status of each study participant for each CF module via interfacing (for automatic exchange between electronic systems) or a graphical user interface (GUI, for user-specific queries). Therefore, a structured version of each participant's CF information is a prerequisite for the electronic use of CF data.

**Figure 1:**
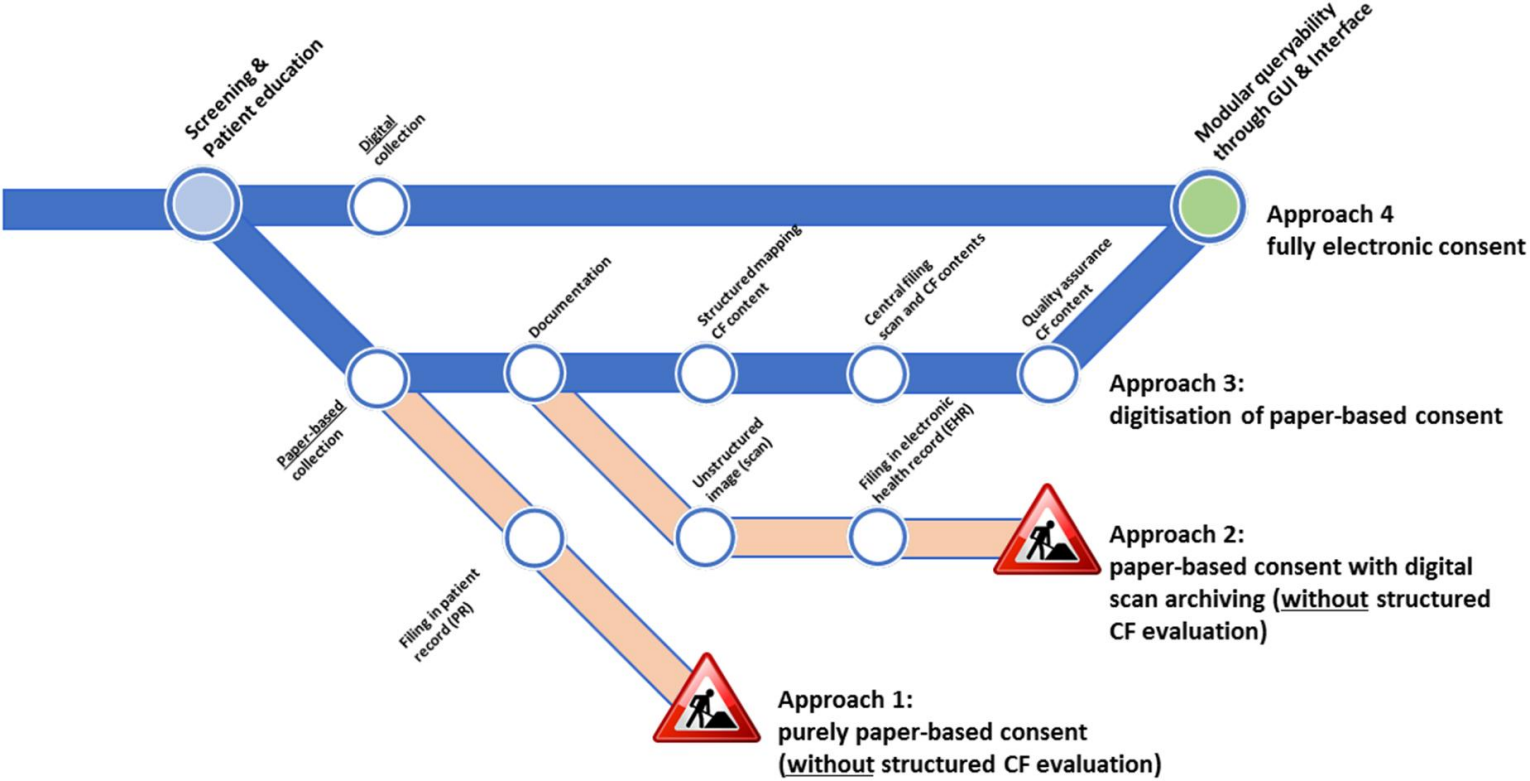
CF collection approaches and their electronic usage suitability. The under-construction icons indicate that structured information could not be obtained without substantial manual efforts. Icon reproduced from “Under construction icon-red” licensed under CC BY 3.0.

Figure 1 illustrates different recruitment workflows with a focus on CF collection and the necessary additional manual work by the study staff who recruit the participants to provide structured and machine-readable consent information.

Recruitment workflows that include CF collection can be classified as follows: (1) a purely paper-and-pencil-based approach, (2) a paper-based approach with digital scan archiving, (3) a digitization approach, and (4) a fully electronic approach. For approaches 1 and 2, paper-based forms are filled in and are either directly filed in a paper-based patient record (approach 1) or are scanned and digitally archived as unstructured images in an electronic health record (approach 2). In both cases, only on-site source data quality assurance measures are possible, and no structured, digitized, and machine-readable information is available. Approaches 3 and 4, as depicted in Figure 1, allow for modular retrievable CF information but differ in the number of steps required. For example, the third approach includes digitization and structuring of information: informed consent is collected on a paper form and then manually documented in an electronic form, which means manually converting the CF form into machine-readable information (manual transcription or using optical character recognition techniques). In addition, the scan of the CF is attached as an unstructured image, and both the structured information and the unstructured image are subsequently used in the central QA measures (e.g., source data verification). As a result, quality-assured, structured, machine-readable, and queryable consent data are available. In the fully electronic approach (approach 4), CFs are filled in via a tablet PC using electronic case report forms. All the information is directly entered by the study staff and participant in a structured way, allowing for immediate plausibility checks. The participant's and the health professional's signatures are also obtained in a digital format. Thus, consent data are provided as structured and machine-readable information upon completion and saving of the CF to the database. Since all the information is shared with the local (patient) information system, no further (manual) work is needed to complete and document the CF of the participant and their consent status.

### 2.4 Recruitment workflow in SÜP and rollout

In SÜP, CFs were collected using approaches 3 (digitization approach) and 4 (fully electronic approach), as illustrated in Figure 1. In both cases, training material on how to document CFs and initial one-on-one training material were provided by the TTP staff.

The core workflow was very similar in both approaches: the clinician filled in the person-identifying information (first and last names, date, and place of birth) on the CF and handed the CF (in a paper form or on a tablet screen) to the eligible person. The potential study participant checked their person-identifying information and corrected it if there were any errors. Furthermore, the potential participant read the provided information, ticked the optional modules “yes” or “no” according to their choice, and signed the CF. Afterward, the CF (in a paper form or on a tablet screen) was returned to the clinician, who also signed the form and stored it (by filing it in the patient's record or by saving it on the tablet, which automatically sent it to the TTP via a secure connection). One copy of the filled-in CF remained with the study participant. In the case of the tablet-based CF, it was printed out after CF completion and provided to the participant.

Paper-based CFs were distributed to each recruiting institution by the SÜP study coordination staff as site-specific printable documents via a web portal. The documents were printed as needed on-site. Digitized CFs were provided as electronic forms by the TTP in the gICS®. The fully electronic consent collection was conducted using tablet PCs and the TTP's gICS® as a secure web application (a personalized TTP safety browser certificate was necessary). Thus, the digital CF collection operated independently of the tablet’s version, operating system, and brand. Therefore, tablet PCs could either be pre-installed with the software and provided by the TTP or the study sites could also use their own existing devices.

### 2.5 Evaluation

To evaluate the impact of the recruitment processes on the study’s inclusion rate and burden on the study staff, the participants who provided their IC via the paper-based digitization approach (Figure 1, approach 3) were used as the control group, because this was the most common way of obtaining consent in the clinical settings of the cohort study analyzed in this article. Those who provided IC via the fully electronic collection approach using a tablet PC (Figure 1, approach 4) were the intervention group. Please note that this is a process evaluation and not a systematic case-control study because the study sites were free to choose whether to collect consent via the paper-based or fully electronic approaches or by using both approaches.

To evaluate the fully electronic CF collection approach compared to the paper-based CF capture procedure, the following criteria were analyzed:

(a) initial CF validity (with/without possible quality issues);
(b) time-to-initial availability of structured consent information (i.e., time lag between recruitment and availability of structured consent information to other systems, such as the LIMS);
(c) time-to-possible-research [i.e., lag between recruitment and possible data usage for (external) researchers] for (i) initially correct CFs and (ii) CFs with initial to-be-corrected quality issues; and
(d) further benefits or disadvantages of each approach according to feedback from study staff and participants to identify strengths and limitations.

Criterion a evaluates the total number of CFs with or without quality issues at initial data entry or digital consent upload. Since CFs are the legal and ethical basis for research, CF quality issues pose a considerable risk to study inclusion rates because the consequence of an invalid CF that is not corrected is study exclusion and, consequently, data deletion. Validity and quality issues are measured with the indicators “completeness” and, for paper-based CFs, “correctness,” according to Nonnemacher et al. (18), and “legal certainty” as defined by the TTP (19). For example, “completeness” (18) describes the degree to which all the optional modules are unambiguously consented to or refused, and all the obligatory fields are filled in. In addition, for the paper-based approach, the QA process investigates whether all the pages of a CF scan are available. As shown in Figure 1, in approach 3, those quality issues fall under the “Documentation” step. “Correctness” is defined as the degree to which all the entered digitized data is concordant with the original paper-based data, i.e., the “Structured mapping of CF content” step in approach 3 being correctly executed, as shown in Figure 1. “Legal certainty” (2, 6, 8) refers to the existence of the signatures of the participant and the clinician on the CF, together with the respective dates of signature. For a complete list of the QA criteria and possible categories of quality issues, see Rau et al. (19).

“Time-to-initial availability of structured consent information” (criterion b) describes the machine-readable availability of structured consent information in other systems, such as an HIS, PACS, and LIMS. This is calculated as the duration between the timestamps for the “original CF signature” and “CF recorded digitally.”

Criterion c, “time-to-possible-research,” evaluates the time needed to confirm quality-assured CFs without quality issues, these being the legal basis to use participants’ datasets for research. This can differ according to the initial quality status (with/without quality issues), and, therefore, was divided into “time-to-possible-research” for (i) initially correct CFs and (ii) CFs with initial quality issues. The time was calculated using the timestamps for “original CF signature” and “successful completion of CF quality assurance.” The measurement units (hours, days, or weeks) of “time-to-usage” and “time-to-research” were determined based on the results. However, the “time-to-possible-research” may have differed from the actual time of availability of the datasets from the SÜP participants to researchers, as further quality criteria independent of consent needed to be observed before the datasets could be made available for research.

The practical usage of paper- or tablet-based CF collection approaches by several medical professions (e.g., data managers, doctors, and study nurses) resulted in feedback provided to the TTP during workshops, subsequent training, or support sessions. Therefore, the benefits and disadvantages of each approach, according to the feedback received from colleagues involved in patient care, were also evaluated for this article as criterion d.

This article only focuses on CFs; thus, withdrawals or other forms of study exclusion besides an invalid CF were not considered.

## 3 Results

### 3.1 Number of consent forms and modes of CF collection used in SÜP

The concept and the processes of IC collection in SÜP were based on the different CFs used in the study. In principle, only CFs that had previously been approved by an ethics committee responsible for the respective site and/or federal state were used at the individual study sites. Therefore, it is possible that only certain versions or a subset of the CFs were available at a particular site. As of July 2024, 63 different versions of SUP-CFs were implemented for 15 federal states and 75 potential recruiting institutions (i.e., registered with the TTP).

Overall recruitment started in November 2020 and ended for COVID-19 patients on 31 July 2023. Control group recruitment ended on 31 December 2023, and follow-ups were possible up until 1 year after infection, i.e., 2024. The fully electronic recording of CFs ceased in June 2023. This was due to the WHO's declaration regarding COVID-19 on 5 May 2023 that lowered its classification from the highest alert to treating and managing COVID-19 like other (endemic) infectious diseases (20). Following this declaration, the number of specialized COVID-19 departments was reduced and the number of new COVID-19 patients at the respective study sites decreased. In the SÜP study, 57 study sites recruited 2,753 study participants (without duplicates and withdrawals) with a total of 3,509 CFs as of 29 July 2024. Among the study sites, 45 (79%) collected paper-based CFs exclusively, while another 11 sites (19%) collected both paper- and tablet-based CFs. Of those 11 sites, three sites collected 85% or more of the CFs via tablets. Only one site (2%) captured all CFs fully electronically using designated tablet PCs. Consequently, the majority of the CFs (3,136, 89.37%) were collected in a paper form, and 373 (10.63%) CFs were captured using the fully electronic approach as of 29 July 2024.

### 3.2 Initial CF quality

**Figure 2:**
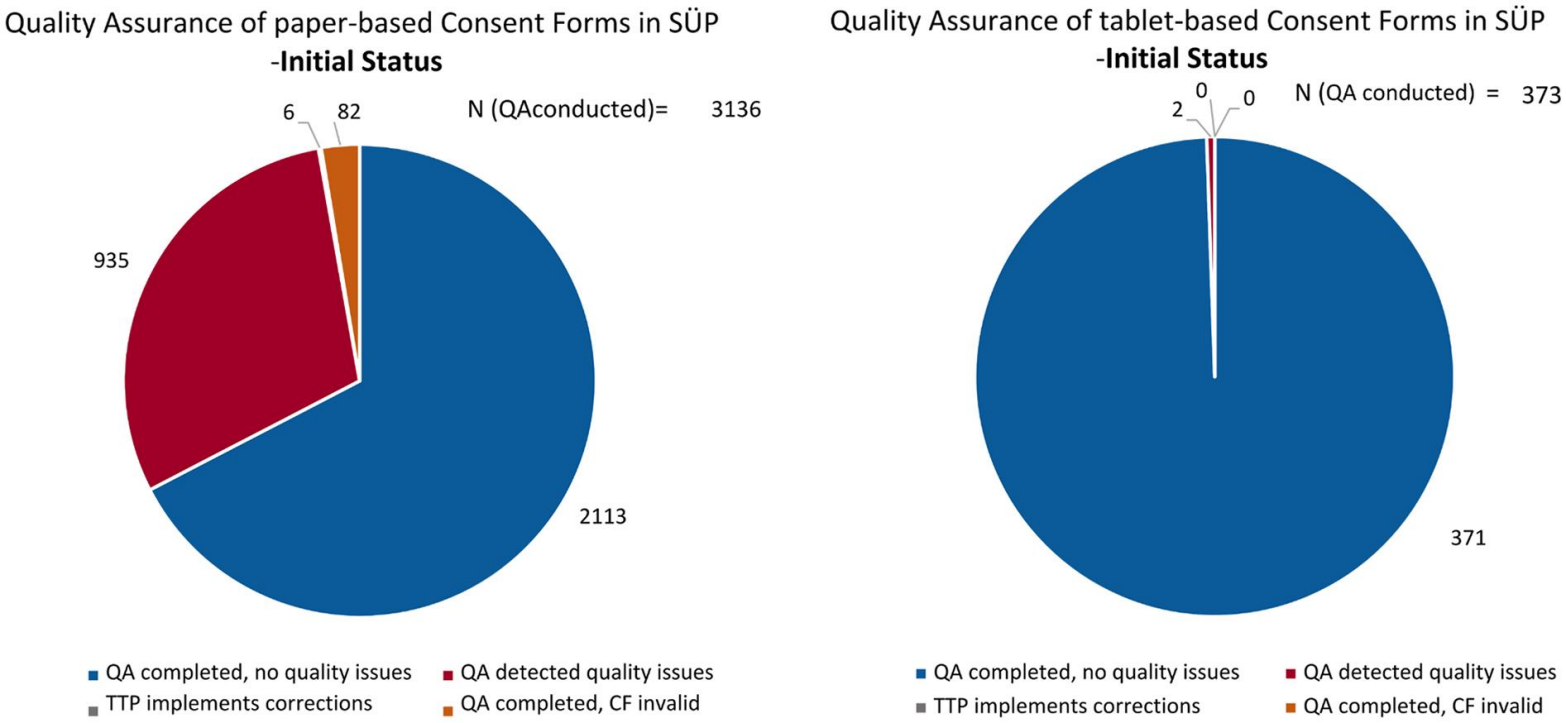
The initial quality, as of 5 May 2022, of the paper-based (left) and fully electronic tablet-based (right) CFs, including the handling of quality issues.

Figure 2 illustrates how the initial CF validity differed between the approaches. Of the CFs that had been quality-assured as of 29 July 2024, 2,113 (67.38%) paper-based and 371 (99.46%) tablet-based CFs were initially valid without any associated quality issues. The remaining 1,023 (32.62%) paper-based CFs and 2 (0.54%) tablet-based CFs showed quality issues and, consequently, were initially invalid.

Interestingly, 501 (15.98%) paper-based CFs showed quality issues related to the documentation process/completeness, and 521 CFs (16.61%) had quality issues related to the structured mapping of CF content/correctness as of July 2024 (see Figure 1, approach 3). Feedback on the quality issues was sent back to the respective study sites with specific requests for correction. In a cohort study, if an invalid CF is not corrected, the corresponding participant's data, including biological samples, must be deleted and are lost to the research.

Most of the quality issues with the paper-based CFs were related to missing scanned pages, missing signatures or dates, or using the wrong CF version. All of these issues were automatically averted in the tablet-based CFs due to the features of electronic data capture. Dates and signatures can be entered easily and are mandatory, i.e., the CF cannot be finalized and saved without entering them. The user is immediately made aware of any missing items by a prompt error message. In addition, correct CF versions are automatically provided, since the study site can only see and choose from their respective valid CF forms. The most common issues with the tablet-based CFs were implausible dates, e.g., the date of the signature is also the participant’s date of birth, or signature fields that were filled in incorrectly [e.g., only a dot or “TN” for “participant” (German: *Teilnehmende*) is provided in the signature field]. Thus, the fully electronic IC collection approach via a tablet prevented the majority of quality issues.

After QA was conducted, 935 CFs were corrected by the study sites, with six CFs requiring internal TTP corrections received from study sites (e.g., changing the electronic consent version). However, 82 (2.62%) paper-based CFs remained invalid (Figure 2).

**Figure 3:**
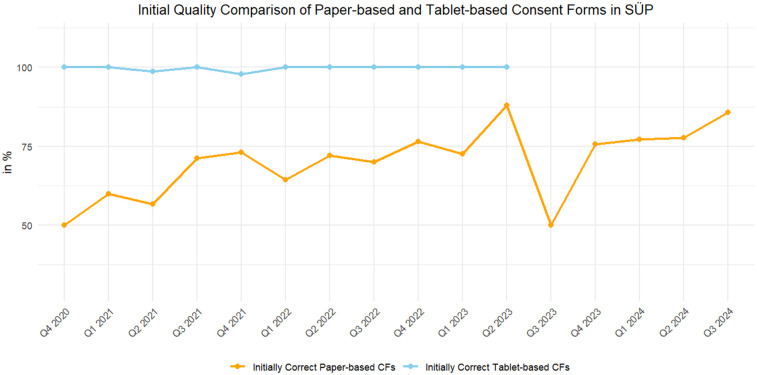
The number of initially valid CFs, as of 29 May 2024, of the paper-based (orange) and fully electronic tablet-based (blue) CFs.

As Figure 3 shows, the initial quality of the paper-based CFs varied greatly over time, whereas the quality of fully electronic CFs remained at a consistently high level.

### 3.3 Unexpected quality issues in the fully electronic CFs

In two cases, human error led to invalid electronic CFs. In the first, the wrong CF version was used, and in the second, the date of birth was incorrectly entered. In the first case, the caregivers of a pediatric patient filled in a CF used for people of legal age instead of a pediatric CF. In this case, the study site provided the correct CF version. The second quality issue occurred during the creation of the participant's record, as the current date was incorrectly used as their date of birth. In this case, the subsequent TTP QA process invalidated the electronic CF, and the study site corrected the CF for the patient.

### 3.4 Time-to-initial availability of structured consent information

The “time-to-initial availability of structured consent information,” calculated from the “original CF signature” and “CF recorded digitally” timestamps, was assessed in days.

A CF can only be used by other systems, such as a HIS, PACS, or LIMS, if it is in a structured digital format. When using tablet-based CFs, which are initially captured as structured digital information, no delay between CF collection and usage by other systems was detected. Therefore, the time-to-initial usage was approximately 0 days for the fully electronic consent form collection approach.

Paper-based forms have to be manually digitized and entered into electronic forms to provide structured information for other systems. Hence, the time-to-initial availability in other systems can include a delay. However, there is no delay (0 days) if the paper-based CF is digitized on the same day as it was signed by the study participant. In total, up to 357 days of delay were registered with a mean delay of 17 days. Long delays were usually detected during periods with high incidence rates. However, the median delay was only 1 day.

### 3.5 Time-to-possible-research

To calculate the “time-to-possible-research,” the difference between the “original CF signature” and “successful completion of CF quality assurance” timestamps was used for the (i) initially correct CFs and (ii) CFs with quality issues, respectively. The results were measured in days.

QA was conducted for all CFs, whether they were newly captured or corrected by the study site. Initially correct CFs can still result in delays if the CF digitization is delayed. The results show that the tablet-based CF collection approach did not result in any time lag (0 days), while the paper-based CF collection approach had a mean delay of 17 days (see previous section).

For CFs with initial quality issues, the main reason for a delay was the time taken to correct the CF at the study site. This usually required waiting for the patient’s next appointment.

Table 1 shows the calculated time-to-possible-research values for paper-based CFs in comparison to tablet-based CFs, with considerably higher values on average for the paper-based CFs. Since the median is less sensitive to outliers, the median value was used for this evaluation criterion.

**Table 1:** The calculated time-to-research values of the CFs with initial to-be-corrected quality issues.

Value	Paper-based CFs (digitization approach), days	Tablet-based CFs (fully electronic approach), days
Minimum	3	0
Maximum	861	0
Mean	84.59	0
Median	48	0

### 3.6 Summary of the evaluation, including the benefits and disadvantages of the approaches according to feedback from study staff and participants

Human factors in the recruitment process and user experiences are major contributors to successful recruitment and study inclusion. The qualitative feedback from clinicians, study staff, and TTP staff regarding the two different recruitment approaches is summarized in Table 2 according to the predefined evaluation criteria and is complemented by the benefits and disadvantages identified in the previous subchapters.

**Table 2:** A summary of the evaluation criteria and the benefits and disadvantages of the two approaches based on qualitative feedback.

Evaluation criteria	Paper-based CFs (digitization approach)	Tablet-based CFs (fully electronic approach)
Initial CF quality		
Initially valid without any quality issues	2,113 (67.38%) CFs	371 (99.46%) CFs
Initial quality issues	1,023 (32.62%)	2 (0.54%) CFs
Time-to-initial availability of structured machine-readable information	Minimum = 0 days	0 days
	Maximum = 357 days	
	Mean = 17 days	
	Median = 1 day	
Time-to-possible-research		
(i) Initially correct CFs	See time-to-initial availability	0 days
(ii) CFs with initial to-be-corrected quality issues	Median = 48 days	Median = 0 days
Further comparisons according to feedback	Easy to use	Easy to use and read, especially due to the user-friendly adjustable font size
	For each CF/version, a sufficient amount of printed paper documents must be kept available at all recruitment sites	Different CFs can be made available and easily chosen, e.g., different languages or simple language versions
	The printed CFs looked very similar, so it is possible that the wrong version could be used	A selection menu with version names is provided to select the required CF version
	An outdated CF version can be filled in when the printed CF is old. As a consequence, the CF is invalid, and a new CF needs to be filled in and signed by the participant	The version provided is always up-to-date, as outdated versions can no longer be filled in
	Common mistakes occur frequently, even after training, and need to be corrected	Common mistakes (e.g., missing items or signatures) are mostly prevented by automatic plausibility checks
	QA requires at least 2 min, including documentation of necessary QA measures	QA is conducted in under 1 min, including documentation of necessary QA measures
	Each paper-based consent form must be manually digitized to obtain structured data after initial consent collection (requiring additional effort), and the scanned image of the CF is not machine-readable	There is immediate availability of structured digital data (no additional efforts are required)
	A high level of effort is required due to post-processing at the TTP and correction at the respective study site	A low level of effort is required at the TTP for QA post-processing; with a medium level of effort required at the study site to edit CF, but this occurs less often
	The paper form needs to be archived (the transport, weight, retrieval, and durability of paper forms could pose problems)	No paper archives are needed, which reduces the workload of the clinicians/study staff
	Available offline	Only available with an Internet connection
		The simple click on “yes” or “no” for optional modules was easier, particularly for people with motor limitations, compared to ticking a designated tick box by hand

## 4 Discussion

MITRE (10) stated in 2014 that collecting CFs digitally is the future. Various commercial software solutions, such as Thieme E-ConsentPro (21), allow researchers to collect electronic CFs without media discontinuity. Therefore, the open-source gICS® software is not the first or only solution to collect CF information for automatic and fully electronic consent management. However, as free-of-charge software for research, it facilitates the translation of informatics solutions and is available to all researchers. Unfortunately, the authors are not aware of any other research evaluating the quality of consent completion using fully electronic approaches compared to paper-based consent collection. Research is usually limited to the structure, content, or information provided in patient information sheets/informed consent forms (22). Therefore, it is a limitation that the findings of this evaluation cannot be compared to other studies’ results.

As stated above, digital consent collection using tablet PCs and the gICS® (12–15) as a secure web application works independently of the tablet’s version, brand, or installed operating system. The study sites decided whether they wanted to use their existing tablet PCs or receive pre-configured TTP tablets. However, practical experience has shown that some types of tablet PCs are more suitable for participants to fill out their consent forms than others due to an optimal screen size, pen recognition, palm rejection, and weight. Although information technology (IT) applications are often seen as barriers for study participation, especially in elderly people, the majority of the SÜP participants provided very positive feedback on and preference for the tablet-based CF, according to the study staff who provided feedback to the TTP. The two main reasons provided were the adjustable font size, which made reading the form easier, and the simple click on “yes” or “no” for optional modules, which was particularly easier for people with motor limitations compared to ticking a designated tick box by hand.

Due to the nature of the gICS® web application (12–15), the electronic CF collection approach required a network connection, rendering offline collection impossible. This drawback was compensated for by using paper-based CFs in the case of a temporarily lost Internet connection. However, from November 2020, this problem only occurred once during SÜP recruitment.

In addition, the electronic consent collection approach lessens the burden on study staff as it makes the provision, digitization, filing, and archiving of paper-based consent forms unnecessary (i.e., it reduces the organizational overheads). Furthermore, the electronic consent collection workflow is still the same for the participant, with basically no need to change routines. Moreover, it streamlines and ends the workflow for clinical staff after the initial patient contact and prevents any typing or transmission errors due to media discontinuity. In particular, when more than one CF version is used, e.g., CFs in different languages, the clinician can easily choose from a given menu with one click instead of being required to select the correct paper forms. As the evidence showed, this one click can also lead to the wrong CF versions being used, e.g., using a consent form for people of legal age instead of one for pediatric patients, leading to unexpected quality issues. However, using the wrong CF version is a human error, which can also happen with paper-based forms. With electronic forms, a software-based plausibility check of the birthdate could lead to a warning message asking the study staff whether they chose the correct CF version for the patient.

Overall, the fully electronic consent collection approach has the potential to reduce inequalities and inequities in healthcare research, as the consent form collection process can be customized according to different requirements. For example, it allows for physically impaired participants to provide informed consent more easily due to adjustable font sizes and larger fields to tick or sign. Since language barriers and a lack of information are possible reasons for the underrepresentation of certain groups (23), the fully electronic consent form collection approach can help overcome these barriers. It allows studies to easily present the information and consent forms in multiple languages to potential participants. It must be noted that regardless of the language used, a native speaker/interpreter is required to explain the content of the consent form and to answer questions in person to ensure that informed consent is given. This person must have medical expertise, i.e., a person accompanying the patient is not sufficient. However, instead of the need to provide printed forms in multiple languages—printed forms are usually limited to an English version and a national language version—one click can change the language of the form according to preference. This enables people to read the information in their native language. Thus, it allows members of ethnic minority groups, who are usually underrepresented in research, to understand the aims of a study and provide informed consent.

Tablet PCs can be used directly at a patient’s bedside without media discontinuity, which is particularly useful in time-critical research areas, e.g., emergency registries or infection research. The rapid availability of the consent status in other systems is essential in such settings. All the NAPKON IT systems had access to the participants’ current consent statuses with no or minimal delay and, for example, indicated whether biological samples could/should be collected for research. In addition, fully electronic consent form collection reduces the time until structured CF data are available. Having data available in real-time allows for data-driven decision-making, a prerequisite for a comprehensive pandemic response and developing future intervention strategies. Since QA of CFs is only one step in the overall QA process, it could only be used to measure “time-to-possible-research.” To also reduce the “time-to-real-research,” electronic CF collection should be integrated into an electronic system with additional plausibility checks for medical data and automatic QA data queries.

When considering the findings regarding the substantial quality difference between the paper- and tablet-based CFs in a major multicentric cohort of SARS-CoV-2-positive patients, it is surprising that the majority of the study sites were consistently recruiting participants solely using paper-based processes. Using the electronic consent collection was a voluntary option and it seems that this was a limitation, with most study staff preferring the usual gold standard of paper-based consent forms. One possible reason is that the study staff assumed that the older the participants were, the less likely it was that they were to use the digital options. However, the evidence has shown that for the aforementioned reasons, the older participants were more willing to use the consent form collection via a tablet. A limitation of this evaluation is that the TTP has, due to its nature, no information about the participants’ medical characteristics. Thus, there is no data on variation among individuals, which could also influence the preference to use paper forms despite having access to the digital consent form. However, it is known that paper-based consent forms were mostly used for inpatients. Because tablets were made available to each site, it can be assumed that there was sufficient hardware available. WLAN availability may be a possible limitation, especially in older buildings. Due to the high number of participating sites, this cannot be clearly determined by the authors. Furthermore, the registration process at some study sites seemed to deviate from the anticipated workflows. At some sites, participants and their identifiable data (IDAT, e.g. first name, surname) were registered at a study workstation and, then, the tablet for consent form collection was brought to and used at the participant's bedside. This could have led to session timeouts if the distances were too far. Therefore, an asynchronous process will be offered in the future, in which participants can be registered with their IDAT and, then, the participant's consent can be obtained via tablet at a later time. In the specific German context of NAPKON, another possible barrier could be that the healthcare professionals felt insecure using electronic forms due to documentation obligations. For example, they may have been unsure whether it was sufficient to collect consent using an electronic form according to national law, or how they could ensure ongoing technical legibility and, thus, feared legal consequences. Since there was no indication of a refusal to fill in tablet-based CFs by participants in SÜP, there are likely other barriers beyond technical availability, user preferences, and workflows. These require further research in a more comprehensive variety of different studies and recruitment settings.

## 5 Conclusion

MITRE's (10) futuristic scenario of collecting CFs digitally has been successfully established and has proven its usefulness in the field of pandemic response with regard to the SÜP COVID-19 study.

Considering the obvious differences between paper- and tablet-based CF collection described in this article, a fully electronic consent collection approach to record consent data in a structured, machine-readable form is the best practice approach. The evaluation in the context of NAPKON's SÜP has shown that tablet-based CFs contributed significantly to improving CF validity and, thus, increased study inclusion and overall recruitment rates. It also streamlined the recruitment process. Moreover, participant acceptance does not seem to be a problem in the tablet-based CF approach.

The next steps include identifying barriers to using the fully electronic CF collection approach among clinicians and further improving the software solution by providing additional functionalities based on users’ feedback, e.g., sending CF copies via email to study participants.

The gICS® software solution (12, 15) is free-of-charge, open-source, and supports the management and querying of structured and semantic consent information via technical standard interfaces (Web-UI, SOAP, HL7 FHIR, and SCC) (13, 24) and, in particular, the new HL7 FHIR consent profiles (25). Researchers and study staff worldwide are welcome to use and integrate the electronic consent collection approach, e.g., using the gICS®, into their workflows, and integrate this fully electronic consent form collection solution into their local and national pandemic response schemes.

## Data Availability

The datasets presented in this article are not readily available because the data (consent forms) underlying this article cannot be shared publicly due to the privacy of individuals (consent forms include sensitive data) who participated in the study. Requests to access de-identified datasets can be directed to the corresponding author.

## Appendix A

The CF contained a total of six optional yes/no questions relating to the following purposes:

- Allowing contact with pre-treating physicians,
- Release from confidentiality for a health insurance company,
- Release from confidentiality for pension insurance,
- Allowing coded transfer of data and biomaterials,
- Allowing re-contact for additional information, and
- Allowing re-contact for feedback on additional medical findings, i.e., incidental findings.

In addition, allowing for biomaterial collection was an optional module if the study site was equipped to collect biomaterial.

## References

[1] Win KT Fulcher JA. Consent mechanisms for electronic health record systems: a simple yet unresolved issue. J Med Syst. (2007) 31(2):91–6. 10.1007/s10916-006-9030-3 17489500

[2] European Medicines Agency. Guideline for Good Clinical Practice E6(R2). London: European Medicines Agency (2018).

[3] World Health Organization, Research Ethics Review Committee (WHO ERC). The process of obtaining informed consent. Available online at: https://www.who.int/docs/default-source/ethics/process-seeking-if-printing.pdf?sfvrsn=3fac5edb_4 (Accessed December 5, 2025).

[4] World Health Organization, Research Ethics Review Committee (WHO ERC). Templates for informed consent forms. Available online at: https://www.who.int/groups/research-ethics-review-committee/guidelines-on-submitting-research-proposals-for-ethics-review/templates-for-informed-consent-forms (Accessed December 5, 2025).

[5] Dierks C Kircher P. Data Privacy in European Medical Research: A Contemporary Legal Opinion. Berlin, Germany: Medizinisch Wissenschaftliche Verlagsgesellschaft (2021).

[6] Regulation (EU) 2016/679 of the European Parliament and of the Council of 27 April 2016 on the Protection of Natural Persons with Regard to the Processing of Personal Data and on the Free Movement of Such Data, and Repealing Directive 95/46/EC (General Data Protection Regulation) (2016).

[7] European Convention on Human Rights, as Amended by Protocols Nos. 11, 14 and 15, Supplemented by Protocols Nos. 1, 4, 6, 7, 12, 13 and 16 (2021).

[8] World Medical Association Declaration of Helsinki: ethical principles for medical research involving human subjects. JAMA. (2013) 310(20):2191–4. 10.1001/jama.2013.281053 24141714

[9] Vogele D Schöffski O Efinger K Schmidt SA Beer M Kildal D. [Analysis of documented informed consent forms for computed tomography: completeness and data quality in four clinics]. Radiologe. (2020) 60(2):162–8. 10.1007/s00117-019-00629-6 31858158

[10] MITRE. Electronic consent management: landscape assessment, challenges, and technology (2014). Available online at: https://www.healthit.gov/sites/default/files/privacy-security/ecm_finalreport_forrelease62415.pdf (Accessed December 5, 202.5)

[11] Schons M Pilgram L Reese JP Stecher M Anton G Appel KS The German National Pandemic Cohort Network (NAPKON): rationale, study design and baseline characteristics. Eur J Epidemiol. (2022) 37(8):849–70. 10.1007/s10654-022-00896-z 35904671 PMC9336157

[12] Rau H Geidel L Bialke M Blumentritt A Langanke M Liedtke W The generic Informed Consent Service gICS®: implementation and benefits of a modular consent software tool to master the challenge of electronic consent management in research. J Transl Med. (2020) 18(1):287. 10.1186/s12967-020-02457-y 32727514 PMC7391490

[13] Bialke M Geidel L Hampf C Blumentritt A Penndorf P Schuldt R A FHIR has been lit on gICS: facilitating the standardised exchange of informed consent in a large network of university medicine. BMC Med Inform Decis Mak. (2022) 22(1):335. 10.1186/s12911-022-02081-4 36536405 PMC9762638

[14] Bialke M Bahls T Havemann C Piegsa J Weitmann K Wegner T MOSAIC—a modular approach to data management in epidemiological studies. Methods Inf Med. (2015) 54(4):364–71. 10.3414/ME14-01-0133 26196494

[15] Trusted Third Party of the University Medicine Greifswald. gICS® (2025). Available online at: https://www.ths-greifswald.de/en/researchers-general-public/gics/ (Accessed December 5, 2025).

[16] Bialke M Penndorf P Wegner T Bahls T Havemann C Piegsa J A workflow-driven approach to integrate generic software modules in a trusted third party. J Transl Med. (2015) 13:176. 10.1186/s12967-015-0545-6 26040848 PMC4467617

[17] Hampf C Geidel L Zerbe N Bialke M Stahl D Blumentritt A Assessment of scalability and performance of the record linkage tool E-PIX® in managing multi-million patients in research projects at a large university hospital in Germany. J Transl Med. (2020) 18(1):86. 10.1186/s12967-020-02257-4 32066455 PMC7027209

[18] Nonnemacher M Nasseh D Stausberg J. Datenqualität in der Medizinischen Forschung: Leitlinie zum Adaptiven Management von Datenqualität in Kohortenstudien und Registern. Berlin, Germany: Medizinisch Wissenschaftliche Verlagsgesellschaft (2014).

[19] Rau H Stahl D Reichel AJ Bialke M Bahls T Hoffmann W. We know what you agreed to, don't we?—Evaluating the quality of paper-based consents forms and their digitalized equivalent using the example of the Baltic Fracture Competence Centre project. Methods Inf Med. (2023) 62(S 01):e10–8. 10.1055/s-0042-1760249 36623832 PMC10306442

[20] World Health Organization. WHO Director-General’s opening remarks at the media briefing—5 May 2023 (2023). Available online at: https://www.who.int/director-general/speeches/detail/who-director-general-s-opening-remarks-at-the-media-briefing—5-may-2023 (Accessed January 8, 2025).

[21] Thieme Compliance GmbH. E-ConsentPro software (2025). Available online at: https://thieme-compliance.de/de/e-consentpro (Accessed December 5, 2025).

[22] Jaramillo Velez AG Aguas Compaired M Granados Plaza M Marino EL Modamio P. [Translated article] Design and validation of two instruments to analyze and evaluate the formal quality in the informed consent process of clinical trials with medicinal products. Farm Hosp. (2023) 47(2):T64–8. 10.1016/j.farma.2023.01.002 36934015

[23] Crittenden-Ward K Micaletto M Olt J Tackett ZC Machizawa S Owuor N An exploration of research participation by persons of minority and underrepresented groups: barriers to diversification in recruitment, minority investigator participation, and solutions. Psychiatry Res. (2022) 308:114333. 10.1016/j.psychres.2021.114333 34952256

[24] Bialke M Hampf C Blumentritt A Moser FM Lang S Stehn A #Consented—a semantic consent code to facilitate consistent documentation and implementation of consent in collaborative medical research. Int J Med Inform. (2024) 190:105545. 10.1016/j.ijmedinf.2024.105545 39018708

[25] German HL7 Working Group. Consent management. FHIR implementation guide (version: 1.0.2) (2023). Available online at: https://ig.fhir.de/einwilligungsmanagement/1.0.2/Home.html (Accessed December 5, 2025).

